# Comparative Analysis of GOCI Ocean Color Products

**DOI:** 10.3390/s151025703

**Published:** 2015-10-12

**Authors:** Ruhul Amin, Mark David Lewis, Adam Lawson, Richard W. Gould, Paul Martinolich, Rong-Rong Li, Sherwin Ladner, Sonia Gallegos

**Affiliations:** 1 BioOptoSense LLC, New Orleans, LA 70115, USA; 2 Naval Research Laboratory, Code 7331, Stennis Space Center, MS 39529, USA; E-Mails: David.Lewis@nrlssc.navy.mil (M.D.L.); Adam.Lawson@nrlssc.navy.mil (A.L.); Richard.Gould@nrlssc.navy.mil (R.W.G.); Sherwin.Ladner@nrlssc.navy.mil (S.L.); Sonia.Gallegos@nrlssc.navy.mil (S.G.); 3 Vencore, Inc., Stennis Space Center, MS 39529, USA; E-Mail: Paul.Martinolich.ctr@nrlssc.navy.mil; 4 Naval Research Laboratory, Code 7231, Building 2, Washington, DC 20375, USA; E-Mail: rong-rong.li@nrl.navy.mil

**Keywords:** geostationary satellite, ocean color remote sensing, bio-optical products, APS, GDPS

## Abstract

The Geostationary Ocean Color Imager (GOCI) is the first geostationary ocean color sensor in orbit that provides bio-optical properties from coastal and open waters around the Korean Peninsula at unprecedented temporal resolution. In this study, we compare the normalized water-leaving radiance (nLw) products generated by the Naval Research Laboratory Automated Processing System (APS) with those produced by the stand-alone software package, the GOCI Data Processing System (GDPS), developed by the Korean Ocean Research & Development Institute (KORDI). Both results are then compared to the nLw measured by the above water radiometer at the Ieodo site. This above-water radiometer is part of the Aerosol Robotic NETwork (AeroNET). The results indicate that the APS and GDPS processed nLw correlates well within the same image slot where the coefficient of determination (r^2^) is higher than 0.84 for all the bands from 412 nm to 745 nm. The agreement between APS and the AeroNET data is higher when compared to the GDPS results. The Root-Mean-Squared-Error (RMSE) between AeroNET and APS data ranges from 0.24 $\left[{\text{mW/(cm}}^{2}\text{srμm})\right]$ at 555 nm to 0.52 $\left[{\text{mW/(cm}}^{2}\text{srμm})\right]$ at 412 nm while RMSE between AeroNET and GDPS data ranges from 0.47 $\left[{\text{mW/(cm}}^{2}\text{srμm})\right]$ at 443 nm to 0.69 $\left[{\text{mW/(cm}}^{2}\text{srμm})\right]$ at 490 nm.

## 1. Introduction

The Geostationary Ocean Color Imager (GOCI) is one of the three payloads of the Korean Communication, Ocean and Meteorological Satellite (COMS), which was successfully launched in June 2010 from the Space Center in Kourou, French Guiana on an Ariane 5 Launch Vehicle [1,2]. GOCI is the world’s first geostationary ocean color sensor designed with visible and near-infrared (NIR) bands that can measure radiance from the ocean surface. GOCI can obtain images every hour during the day, which is ideal to monitor short-term temporal variability of the ocean in near real time. GOCI covers a 2500 × 2500 km square around the Korean Peninsula centered at 36°N and 130°E with an approximate 500 m pixel size, and it is comprised of sixteen (4 × 4) slot images [1,2]. It also has high signal-to-noise ratio (over one thousand) which is necessary for detection of low and rapidly varying reflectance signals. GOCI has six visible bands centered at 412 nm, 443 nm, 490 nm, 555 nm, 660 nm and 680 nm, and two NIR bands with bands centered at 745 nm and 865 nm for atmospheric correction. 

Polar-orbiting satellite sensors such as the Moderate Resolution Imaging Spectroradiometer (MODIS) and the MEdium Resolution Imaging Spectrometer (MERIS) have been widely used for ocean color studies [3,4]. Those sensors typically collect data at about 1 km resolution and one image per day. While these sensors provide an enormous advantage for global spatial coverage, cloud-contaminated pixels can seriously restrict the number of valid ocean retrievals. About two-thirds of the Earth’s surface is always covered by clouds [5] which also cast shadows that contaminate ocean color pixels [6,7]. Daily revisit time also precludes monitoring of the dynamic ocean variability at hourly time scales, which is particularly important in optically-complex coastal waters that change quickly due to tides, river discharge, wind-driven advection, and bottom re-suspension. For these reasons, a geostationary sensor with high temporal frequency is ideal for studying and quantifying biological and physical processes in the coastal ocean. Unlike polar-orbiting satellites, which provide only one or two images of the same geographic area per day, GOCI collects images every hour from 00:16 UTC to 07:16 UTC (total eight images per day). This high-frequency image acquisition enables more detailed time-series analyses to detect and monitor phytoplankton blooms [8,9], sediments, and river discharge plumes [9], thereby aiding development and testing of short and long term predictive models. 

Accurate estimation of ocean color products is important for primary production models, carbon budgets, hypoxia, and eutrophication, among others. Remote sensing has opened an effective way to estimate these products. The National Aeronautics and Space Administration (NASA) Earth Observing System (EOS) and European Remote Sensing (ERS) programs provide ready-to-use remote sensing products that are generated by science-based, calibrated, and validated algorithms [10,11]. The fundamental measurement in ocean color remote sensing is the water-leaving radiance (Lw), the upwelling spectral distribution of the radiance from the ocean. Geophysical parameters such as chlorophyll can be retrieved from this water-leaving signal since it contains information about the optically-active components in the water column. However, only about 10% of the total signal measured by the ocean color sensors contains information about the waters; the rest represents scattering from aerosols and air molecules [12]. The goal of the atmospheric correction over the ocean is to remove contributions from the atmosphere and sea-surface reflection from the top-of-atmosphere (TOA) radiance recorded by the sensor, yielding Lw. However, differences exist in these ready-to-use standard products due to sensor characteristics, atmospheric corrections, and product generation algorithms [13]. 

The Automated Processing System (APS) was developed by the Naval Research Laboratory (NRL) at Stennis Space Center (NRL/SSC) to produce daily ocean color products from real-time or archived Advanced Very High Resolution Radiometer (AVHRR), Sea-Viewing Wide Field-of-View Sensor (SeaWiFS), MODIS, MERIS, Visible Infrared Imaging Radiometer Suite (VIIRS), Hyperspectral Imager for the Coastal Ocean (HICO), and GOCI satellite imagery [14]. It is a powerful, extendable, image-processing tool, which is a complete end-to-end system that includes sensor calibration, atmospheric correction, and bio-optical inversion. The APS level-2 program n2gen is an extension of NASA’s l2gen obtained from SeaWiFS Data Analysis System (SeaDAS) code. It produces NASA-standard ocean color products, as well as operational Navy-specific products using NRL algorithms [14]. APS operates in stand-alone batch processing mode, which facilitates testing and validation of new products and algorithms, and reprocessing of many data files (dozens of scenes/day). Furthermore, APS can automatically extract image data from a region-of-interest (ROI) to facilitate time-series analyses and also match-ups with *in situ* data for a specific location. NRL/SSC is one of only a few institutions that has implemented the complete MODIS, VIIRS, and GOCI processing codes while maintaining compatibility with NASA/Goddard SeaDAS products. 

The standard data processing software system for GOCI data consists of the Image Processing System (IMPS) and the GOCI data processing system (GDPS) [15,16]. The IMPS is used to generate level 1B data from raw data while GDPS is used with level 1B data to generate processed level 2 data which consist of ocean color products such as Lw, normalized water-leaving radiance (nLw), chlorophyll concentration, and total suspended sediment (TSS) concentration. GDPS also generates level 3 data which consist of fishery information, primary productivity, water quality level and water current vectors [17]. 

For sensor inter-comparison or merging of their data, and in order to maintain consistency and reduce errors, it is important to process all the data with the same processing system and to maintain vicarious calibration gain sets generated using the same standard calibration and validation site. The polar-orbiting satellites (ex. MODIS and VIIRS) are vicariously calibrated using the Marine Optical BuoY (MOBY) located off the coast of Hawaii. GOCI vicarious calibration gains were generated using a clear water site selected in the Japan Sea and MODIS Aqua data as the calibration source. The goal of this study is to compare APS processed GOCI results with those obtained by running the stand-alone GDPS code. Both satellite results are statistically validated with coincident Aerosol Robotic NETwork (AeroNET) measurements [18,19].

## 2. Data

The nLw measurements were extracted from the AeroNET sensors using the Satellite Validation Navy Tool (SAVANT), a web-based tool developed at NRL/SSC [20,21]. In this system, APS satellite derived georeferenced daily nLw is automatically extracted at ROI and inserted into the Structured Query Language (SQL) database. SAVANT can also query the database for coincident *in situ* or AeroNET data and automatically conduct a vicarious calibration if both the satellite and *in situ* or AeroNET data are valid. Vicarious calibration can use *in situ*, AeroNET, or other sensor data to calibrate the TOA radiance (Lt) measurement of the sensor [22,23,24]. During the conversion of the Level 1B files (Sensor Data Record) to Level 2 (Environmental Data Record), the atmospheric correction process is performed to generate nLw and remote sensing reflectance ($R_{rs}$) values. Requested products can then be generated from the nLw and $R_{rs}$ spectra. While the selected atmospheric correction is being performed, various scattering and absorption factors are generated for use in adjusting the Lt value to derive the nLw value for each wavelength band. These include Rayleigh and aerosol scattering coefficients and atmospheric gas absorption coefficients. After the satellite-derived nLw values have been computed, the various scattering and absorption factors are still held in memory. If known *in situ* nLw data is available, the satellite-derived nLw values can then be replaced by the *in situ* nLw values. All the atmospheric correction scattering and absorption factors held in memory can then be added to the *in situ* nLw values. This results in a vicarious TOA radiance measurement, vLt, for each wavelength band. Vicarious calibration is a process that uses *in situ* water radiance measurements coincident with the sensor overpass to correct Lt values measured by the sensor. The vicarious calibration performs this correction by adding the *in situ* water radiance measurements to the atmospheric radiance terms resulting in a “vicarious” TOA radiance (vLt) value. The ratio of the vLt/Lt provides a vicariously-calibrated gain factor that, when multiplied to the Lt value, provides the TOA radiance value needed to derive the *in situ* nLw values as the satellite-derived nLw measurements. The Level 1B data can be reprocessed while using the newly computed gains. Root-mean-square (RMS) errors can be generated between the new satellite-derived nLw values and the *in situ* nLw values and compared to RMS errors generated before the application of the new gains in order to assess the improvement in the measurement of nLw [22].

The AeroNET station closest to the Korean Peninsula is the Ieodo AeroNET site, and it represents typical oceanic conditions off the island of Ieo (Ieodo) in the East China Sea at latitude 32.123°E and longitude 125.1824°N. Ocean color measurements, including nLw from the Ieodo site, are not collected as frequently as at other AeroNET sites, such as the Acqua Alta Oceanographic Tower (AAOT) or the Wave-Current-Surge Information System (WavCIS). Thus, only a limited number of AeroNET nLw measurements were available at this location for comparison to the GOCI satellite imagery. We acquired all the nLw data from the Iedo AeroNET site from January 2013 to June 2014 through SAVANT. After proper quality control imparted by NASA’s Ocean Biology Processing Group (OBPG) [25], there were only 13 Ieodo AeroNET measurements coincident with the available GOCI data. For the matchup, we programmed SAVANT to extract GOCI satellite data acquired within ±3 h of the AeroNET measurements. This time window allowed 17 valid APS processed GOCI pixel data. For the stand-alone GDPS processed data, only 13 of the 17 coincident pixels were valid. Although AeroNET nLw spectra from 11 March 2014 passed the quality control, the spectra did not look like a typical water spectra. This could be due to a number of reasons including post processing glint correction failure, cloud shadows or cloud edges. Sometimes these issues only can be noticed in the resulting spectra. There were two valid GOCI acquisitions (05:16 UTC and 07:16 UTC) within ± 3 h of this particular AeroNET measurement where APS-GOCI and GDPS-GOCI retrieved nLw agreed well with each other. GDPS GOCI processed nLw spectra at the Ieodo pixel from 15 April 2014 exhibited negative values at the red wavelengths. Therefore, we excluded this matchup as well. After all the exclusions, there were 10 coincident AeroNET, APS-GOCI, and GDPS-GOCI processed results available for the comparative analyses. Table 1 summarizes AeroNET and coincident satellite measurements. 

**Table 1:** Description of coincident AeroNET and GOCI ocean color data. For each date, the acquisition time (UTC) is indicated in hh:mm:ss.

Date	Time (GOCI)	Time (AeroNET)	Time Difference (in minute)
12/07/2013	02:15:37	02:03:12	12
01/23/2014	03:15:36	02:25:14	50
01/27/2014	04:15:37	05:26:13	71
01/27/2014	06:15:37	05:26:13	49
03/16/2014	05:15:37	06:59:26	104
03/16/2014	07:15:37	06:59:26	16
04/07/2014	06:15:35	05:48:52	27
04/09/2014	01:15:36	23:42:47 *	93
04/09/2014	03:15:36	05:14:45	119
04/09/2014	05:15:36	05:14:45	01

* represents data acquired on 04/08/2014.

We acquired all 10 coincident GOCI L1B images for the dates shown in Table 1 and also all 8 hourly GOCI L1B images from 5 April 2011 through the Naval Oceanographic Office (NAVOCEANO). All GOCI L1B data were processed through APS v5.8 and GDPS v1.2 to compute level-2 ocean color products, including nLw at all visible and NIR bands. Note that *nLw* is the fundamental measurement in ocean color remote sensing that contains information of the optically-active components of the water column. 

## 3. Results and Discussion

To achieve accurate nLw, a vicarious calibration was implemented, such calibration of the TOA signal through ground-truth measurements, can be applied to satellite ocean color data [22,23,24]. Recently, NOAA has released a series of calibration coefficients (gains) for the GOCI sensor, generated from MODIS data. Crout *et al*. [26], demonstrated improved ocean color retrievals with these gains. The gains have been applied to all APS processed imagery in this study. GDPS was vicariously calibrated with data from MODIS, MERIS, and some *in situ* measurements in Korean waters [16]. 

An aerosol model needs to be selected for each pixel in a scene to provide the aerosol scattering information. Aerosol models characterize the aerosol radiance and scattering associated with different relative humidity and particle size distributions. Although other methods are available within APS, the current study uses the aerosol model selection process developed by Gordon and Wang [27]. This process computes an aerosol parameter based on the ratio of the single scattering reflectance between two NIR bands. A NIR band ratio has also been generated for each of the aerosol models specifically characterizing the aerosol scattering exhibited by atmosphere with the model’s specific relative humidity and particle size distribution. The aerosol model with an associated NIR reflectance ratio that most closely matches the computed NIR reflectance ratio from the sensor scene pixel is selected as the aerosol model to use. In many cases the closest two models are selected and interpolation between the two is performed to generate the final aerosol scattering estimate. APS has 80 aerosol models from which to make its aerosol model selection [14] while GDPS computes aerosol scattering differently [15,16]. Therefore, some discrepancies in the results may arouse from the differences in aerosol scattering estimation. 

### 3.1. AeroNET vs. Satellite

**Figure 1:**
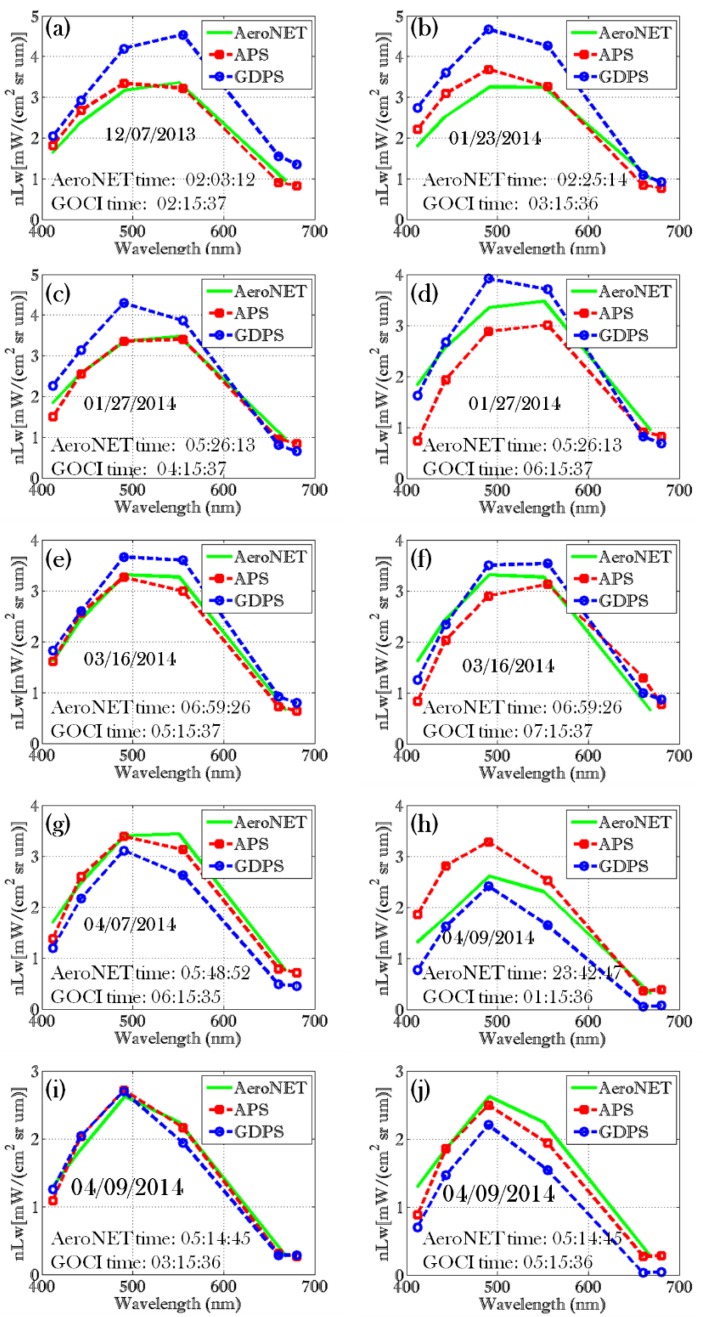
Spectral comparison of nLw at the AeroNET pixel: AeroNET spectra (green), APS retrieved spectra (red), and GDPS retrieved spectra (blue). (**a**) 12/07/2013 GOCI image acquired at 02:15:37 UTC; (**b**) 01/23/2014 GOCI image acquired at 03:15:36 UTC; **(c**) 01/27/2014 GOCI image acquired at 04:15:37 UTC; (**d**) 01/27/2014 GOCI image acquired at 06:15:37 UTC, (**e**) 03/16/2014 GOCI image acquired at 05:15:37 UTC; (**f**) 03/16/2014 GOCI image acquired at 07:15:37 UTC; (**g**) 04/07/2014 GOCI image acquired at 06:15:35 UTC; (**h**) 04/09/2014 GOCI image acquired at 01:15:36 UTC; (**i**) 04/09/2014 GOCI image acquired at 03:15:36 UTC; and (**j**) 04/09/2014 GOCI image acquired at 05:15:36 UTC.

To evaluate the performance of the APS processed GOCI data, we compared APS and GDPS derived nLw at the Ieodo pixel with AeroNET nLw values. Although GOCI and AeroNET have somewhat different spectral bands, it is possible to select a set of comparable bands centered at 412 nm, 443 nm, 490 nm, and 555 nm for comparison purposes and for quantitative analyses. For the qualitative spectral evaluation, we use the AeroNET band at 671 nm with GOCI bands 660 nm and 680 nm. All the data were quality controlled using standard ocean color flags such as cloud mask, land mask, atmospheric correction failure, and high glint to filter out bad data. Spectral comparison between AeroNET, APS results and GDPS results are shown in Figure 1. AeroNET spectra are shown in green, APS spectra in red, and GDPS spectra in blue. Considering the differences due to natural variability in the spatial resolution among the AeroNET “point” measurement, the 500 m GOCI pixel, and the uncertainties in satellite data processing (geolocation accuracy and atmospheric correction in particular), the overall agreement between the AeroNET and satellite data is fairly close. 

For the quantitative analyses, we calculated:

(1) the root-mean-square error (RMSE):

$$RMSE=\sqrt{\frac{\sum_{i=1}^{i=N}{\left(nLw_{AeroNET}(i)-nLw_{GOCI}(i)\right)}^{2}}{N}}$$

and (2) the mean absolute percentage difference (APD in %):

$$APD=100\frac{1}{N}\sum_{i=1}^{i=N}\frac{\left|nLw_{AeroNET}(i)-nLw_{GOCI}(i)\right|}{nLw_{AeroNET}(i)}$$

where, $nLw_{AeroNET}(i)$ and $nLw_{GOCI}(i)$ indicates AeroNET nLw and GOCI nLw value of the *i*-th matchup, respectively, and *N* is the total number of the matchup pairs. Note that there are two sets of GOCI data (APS processed and GDPS processed). Thus, we estimate two sets (AeroNET *vs*. APS and AeroNET *vs*. GDPS) of RMSE and APD values. For all 10 matchup shown in Figure 1, RMSE between AeroNET and APS results ranges from 0.24 $\left[{\text{mW/(cm}}^{2}\text{srμm})\right]$ at 555 nm to 0.52 $\left[{\text{mW/(cm}}^{2}\text{srμm})\right]$ at 412 nm while RMSE between AeroNET and GDPS results ranges from 0.47 $\left[{\text{mW/(cm}}^{2}\text{srμm})\right]$ at 443 nm to 0.69 $\left[{\text{mW/(cm}}^{2}\text{srμm})\right]$ at 490 nm. Similarly the APD values between AeroNET and APS results ranges from 26.84% at 412 nm to 6.79% and 555 nm while APD between AeroNET and GDPS results ranges from 26.72% at 412 nm to 15.91% at 443 nm. RMSE and APD values are summarized in the Table 2. For this dataset, overall agreement between AeroNET and APS results higher RMSE and APD than the AeroNET and GDPS results. This may be the result of the difference in atmospheric correction algorithms or simply the version of the GDPS software available to us at the time of the investigation. Additionally, the dataset used in this study is rather small. A larger data set over different types of water is necessary for a full comparison. 

**Table 2:** RMSE $\left[{\text{mW/(cm}}^{2}\text{srμm})\right]$ and APD (%) between AeroNET, APS, and GDPS results at the Ieodo location.

AeroNET *vs*. APS *vs*. GDPS	nLw(412)	nLw(443)	nLw(490)	nLw(555)
AeroNET *vs*. APS				
RMSE	0.52	0.46	0.33	0.24
APD	26.84%	15.86%	8.23%	6.79%
AeroNET *vs*. GDPS				
RMSE	0.49	0.47	0.69	0.67
APD	26.72%	15.91%	17.24%	19.92%

### 3.2. APS vs. GDPS Results

**Figure 2:**
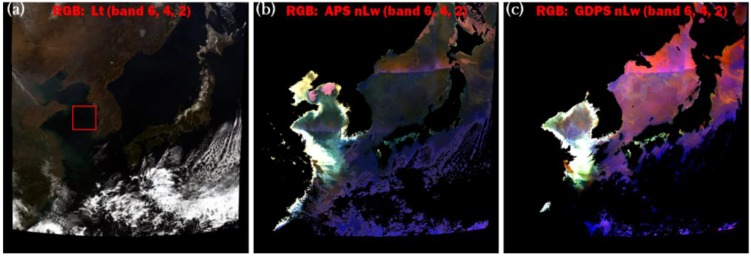
GOCI scene collected at 03:16 UTC on 5 April 2011: (**a**) true color image created using the TOA total radiance (Lt); (**b**) true color image created using APS processed (atmospherically corrected) nLw; and (**c**) true color image created using GDPS processed (atmospherically corrected) nLw.

As the data at the AeroNET Ieodo location, all the satellite data processed through APS and GDPS were quality controlled using standard flags. We noticed that the GDPS flags out significantly more pixels than the APS. Many of the pixels that are flagged out by GDPS but not by APS appear to be cloud free on the TOA true color images. To confirm this was the case, we created true color (R, G, B) images for the whole GOCI coverage area using band 6 (680 nm) for the red channel (R), band 4 (555 nm) for the green channel (G), and band 2 (443 nm) for the blue channel (B). Three true color images were created for the GOCI scene collected at 03:16 UTC on 5 April 2011. Figure 2a was created using the TOA total radiance (Lt), Figure 2b was created using APS processed (atmospherically corrected) nLw, and Figure 2c was created using GDPS processed (atmospherically corrected) nLw. Clouds, lands, and other invalid pixels are shown in black in both atmospherically corrected true color images (Figure 2b,c). Inspection of these two figures reveals a discontinuity in color along a bright straight line in the upper half of both atmospherically corrected images. This is noticeable in the Sea of Japan. The discontinuities occur at places where GOCI slot images are stitched together. The GOCI data is composed of sixteen (4 × 4) slot images where time difference between slot-1 and slot-16 is about 30 min [15,16]. GDPS corrects for the slot image time difference while APS currently does not. Due to the processing differences, we did not extract data across image slot overlapping regions. 

It can be clearly seen that while the APS image has the same cloud spatial coverage as the TOA true color image (Figure 2a), the GDPS true color image (Figure 2c) has many more black pixels than the APS image (Figure 2b). In fact, GDPS over corrects for clouds and often flags turbid waters as potential clouds. This is consistent with the findings from a previous study [28], where GDPS processed GOCI results were compared with MODIS and MERIS results. Note that APS maintains consistency with MODIS, thus this finding complements the previous study [28]. To quantify the number of valid pixels through APS and GDPS, we acquired all eight GOCI images (00:16 UTC to 07:16 UTC) from 5 April 2011. However, to reduce errors due to viewing and illumination geometry, we excluded the first two images (00:16 UTC and 01:16 UTC) and the last two images (06:16 UTC and 07:16 UTC). The remaining four hourly images, acquired from 02:16 UTC to 05:16 UTC, were cloud free over a significant portion of slot image-7. We selected a ROI in this slot (shown with a red box in Figure 2a) for further analysis. The selected area contains 230,231 GOCI pixels covering both coastal and open-water regions. Our analysis indicates that even though the box is nearly cloud free, APS retrieves 2%–6% more valid pixels than the GDPS (See Table 3) due to more failures during GDPS processing. 

**Table 3:** Slopes (S), intercepts (I), and coefficient of determination (r^2^) of regression analyses obtained between APS and GDPS processed nLw. Regression analyses were performed on the GOCI images acquired on 5 April 2011 at 02:16 UTC, 03:16 UTC, 04:16 UTC, and 05:16 UTC. Each regression was established for the ROI shown in Figure 2a with the red box containing a total 230,231 pixels (before quality control) for each image.

Acquisition Time	# of Valid Pixels APS	# of Valid Pixels GDPS	# of Valid	nLw(412)	nLw(443)	nLw(490)	nLw(555)	nLw(660)	nLw(680)	nLw(745)
02:16 UTC	228,015 (2.83% more valid pixels)	221,495	221,492	r^2^ = 0.84	r^2^ = 0.95	r^2^ = 0.99	r^2^ = 0.99	r^2^ = 0.99	r^2^ = 0.99	r^2^ = 0.99
				S = 0.72	S = 0.85	S = 0.94	S = 0.96	S = 1.00	S = 0.97	S = 0.75
				I = 0.75	I = 0.50	I = 0.37	I = 0.07	I = 0.04	I = 0.02	I = 0.00
03:16 UTC	228,541 (2.10% more valid pixels)	223,709	223,708	r^2^ = 0.91	r^2^ = 0.97	r^2^ = 0.99	r ^2^ = 0.99	r^2^ = 0.99	r^2^ = 0.99	r^2^ = 0.99
				S = 0.80	S = 0.91	S = 0.99	S = 0.99	S = 1.04	S = 1.00	S = 0.77
				I = 0.67	I = 0.39	I = 0.24	I = −0.02	I = -0.02	I = −0.04	I = −0.01
04:16 UTC	228,936 (2.00% more valid pixels)	224,338	224,337	r^2^ = 0.92	r^2^ = 0.98	r^2^ = 0.99	r^2^ = 0.99	r^2^ = 0.99	r^2^ = 0.99	r^2^ = 0.99
				S = 0.84	S = 0.93	S = 1.00	S = 1.01	S = 1.04	S = 1.00	S = 0.75
				I = 0.84	I = 0.57	I = 0.39	I = 0.08	I = 0.05	I = 0.03	I = 0.00
05:16 UTC	229,027 (5.93% more valid pixels)	215,369	215,368	r^2^ = 0.86	r^2^ = 0.96	r^2^ = 0.98	r^2^ = 0.99	r^2^ = 0.97	r^2^ = 0.97	r^2^ = 0.96
				S = 1.10	S = 1.09	S = 1.11	S = 1.09	S = 1.10	S = 1.05	S =0 .76
				I = 0.54	I = 0.28	I = 0.14	I = −0.08	I = −0.02	I = −0.03	I = −0.01

**Figure 3:**
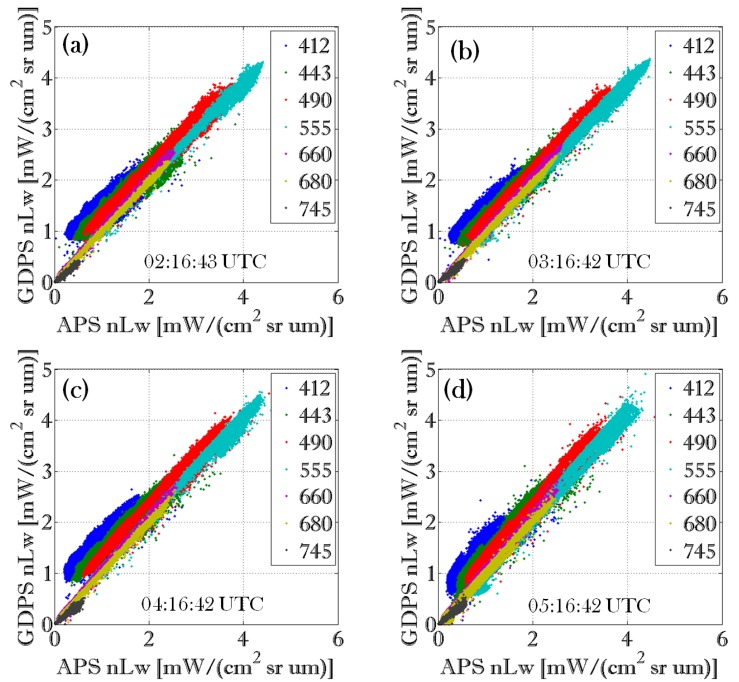
Scatterplots between APS and GDPS datasets from 5 April 2011 for all GOCI ocean color bands including NIR: (**a**) for the image acquired at 02:16:43 UTC; (**b**) for the image acquired at 03:16:42 UTC; (**c**) for the image acquired at 04:16:42 UTC; and (**d**) for the image acquired at 05:16:42 UTC.

For further quantitative inter-comparison of the of APS and GDPS results, we analyzed nLw values from the ROI shown in Figure 2a where both APS and GDPS pixels are valid. Scatterplots from the 5 April 2011 scene between APS and GDPS datasets for all GOCI ocean color bands including NIR are shown in Figure 3. Figure 3a corresponds to the image acquired at 02:16 UTC, Figure 3b to the image acquired at 03:16 UTC, Figure 3c to the image 04:16 UTC, and Figure 3d to the image acquired at 05:16 UTC. Table 3 shows that the correlation between the two datasets is quite high, with the lowest coefficient of determination (r^2^) of 0.84 observed for the 412 nm band of the GOCI data acquired at 02:16 UTC; the r^2^ was nearly 0.99 for most of the other bands from 490 nm to 745 nm for all four images. Although the slopes and intercepts deviate somewhat from the 1:1 line in the blue-green wavelength regions (as can be seen in Figure 3 and Table 3), they improved at higher wavelengths. This may be the result of the differences in aerosol correction during atmospheric correction process since APS and GDPS selects aerosol model differently. Aerosol signal is much stronger in the shorter wavelength, thus larger errors are expected at shorter wavelength during extrapolation using NIR bands [13]. 

## 4. Conclusions

The results of the qualitative and quantitative analyses indicated differences in the GOCI products generated by the APS and the GDPS software. However, due to the limited number of observations, we cannot easily indicate that these differences are statistically significant. We can, however, speculate that factors that may have contributed to the differences include atmospheric correction procedures, glint correction, number of aerosol models used for the atmospheric correction. The authors recommend that future studies be conducted with much larger datasets, and with the latest versions of both software packages to obtain statistically significant results. This study illustrates the need for software packages processing the same ocean color data to have the same functionality in order to obtain comparable results.
